# Comparing the hydrological performance of an irrigated native vegetation green roof with a conventional *Sedum* spp. green roof in New York City

**DOI:** 10.1371/journal.pone.0266593

**Published:** 2022-04-20

**Authors:** Nandan H. Shetty, Robert M. Elliott, Mark Wang, Matthew I. Palmer, Patricia J. Culligan

**Affiliations:** 1 Department of Civil and Environmental Engineering, The Citadel, Charleston, South Carolina, United States of America; 2 Department of Civil Engineering and Engineering Mechanics, Columbia University, New York, New York, United States of America; 3 Department of Ecology, Evolution, and Environmental Biology, Columbia University, New York, New York, United States of America; 4 Department of Civil and Environmental Engineering and Earth Sciences, University of Notre Dame, Notre Dame, Indiana, United States of America; Georgia Southern University, UNITED STATES

## Abstract

The objective of this study was to compare the hydrological performance of an irrigated, 127 mm deep green roof, planted with vegetation native to the New York City area, to a conventional, non-irrigated, 100 mm deep green roof, planted with drought-tolerant *Sedum* spp. Four years of climate and runoff data from both green roofs were analyzed to determine seasonal stormwater retention. Empirical relationships between rainfall and runoff were developed for both roofs, and applied to historical rainfall data in order to compare stormwater retention values for different rainfall depths. Crop coefficients for the vegetation on each green roof were estimated using the soil moisture extraction function. This function was also used to estimate monthly evapotranspiration. Despite being irrigated, the green roof with native vegetation retained more stormwater per annum (64%) than the non-irrigated green roof planted with *Sedum* spp. (54%). The green roof planted with native vegetation also had approximately twice the crop coefficient (1.13) than the green roof planted with *Sedum* spp. (0.57), indicating that the New York City native plants transpire more stormwater than the *Sedum* spp. plants given certain climate and substrate moisture conditions. Overall, the results of the study indicate that, for the New York City climate region, irrigated green roofs of native vegetation have the capacity to better manage stormwater than non-irrigated green roofs planted with drought-tolerant succulents.

## Introduction

Urban stormwater runoff from impervious surfaces reduces water quality and ecological diversity in surrounding water bodies [1]. Because rooftops represent about half of the impervious surface in some cities [2, 3], they are a high priority for interventions that improve urban stormwater management. Vegetated roofs, known as green roofs, cool roofs, eco roofs, roof gardens, or living roofs [4], can annually retain 30% to 86% of rainfall [5], making them an attractive strategy for reducing rooftop runoff. Because of varying environmental conditions and the lack of generally accepted national standards for green roofs, the vegetation and configuration of green roofs in the U.S. can vary greatly [6].

Green roofs are prone to more extreme weather conditions than natural habitats on the ground; they are colder in the winter, hotter in the summer, and more susceptible to rapid desiccation [7]. As a result, green roofs are typically planted with hardy desert vegetation such as *Sedum* spp. [8, 9]. *Sedum* spp. are drought-tolerant plants, adapted to a hot, dry climate, and have low mortality rates in harsh rooftop conditions [2, 8, 10]. The fact that most *Sedum* spp. are not native to North America, however, has motivated some American green roof designers to seek alternative options. As a result, the use of plants native to a given climate is currently being explored to understand their ability to perform under the harsh climate of exposed green roofs [8]. Programs such as the Sustainable Sites Initiative certification program, which was created by the United States Green Building Council, are encouraging this exploration by awarding points for green roof projects that include native plants [7].

The category of ‘native plant’ may seem vague; it is however the term used by many specialists [7]. This is because plants adapted to a specific climate are necessarily particularly suited to its temperature and rainfall, and the plants native to the New York City (NYC) region are consequently acclimated to NYC’s temperate, relatively humid climate. They have physiological characteristics that may confer many advantages to treating the abundant stormwater in NYC. For example, while drought-tolerant succulent plants such as *Sedum* spp. use crassulacean acid metabolism (CAM) photosynthesis, thereby minimizing water loss by only allowing transpiration to occur at night (stomata are kept closed during the day, restricting the total volume of water that a plant may transpire), most species native to NYC do not employ CAM photosynthesis. Instead, they transpire more water in between rainfall events, leaving more unsaturated space in a green roof’s substrate for stormwater retention [11]. Similarly, the height of plants native to the NYC region may confer advantages over low-lying *Sedum* spp. (see Discussion). Finally, these native plants display a diversity of root and shoot structures, including more tap roots, fibrous roots, and leaf hairs that provide different absorption and transpiration of water compared to succulents. This diversity may lead to better stormwater management overall [9]. It is likely for these reasons that green roofs planted with varied, native vegetation manage more stormwater than roofs planted with *Sedum* spp., as many studies have found [5, 11–13].

Still, questions remain, as *Sedum* spp. do certainly have their own advantages when it comes to thriving on manmade green roofs. While the studies cited above found that using native vegetation on green roofs increased stormwater retention, others found no significant differences in hydrologic performance between *Sedum* spp. and native plants [8, 14–16]. In fact, Stovin et al. [14] reported that *Sedum* spp. have greater evapotranspiration (ET) and helped retain more stormwater runoff than a treatment of native vegetation. A perceived uncertainty in the performance behavior of native versus *Sedum* spp. green roofs might be one reason why many engineers remain hesitant to promote native plants, in contrast to the general trend among architects, landscape architects, and biologists, who tend to favor native plants [7]. Another reason might be the irrigation requirements for native vegetation. Irrigation improves plant survival and increases the range of plant species capable of surviving harsh rooftop conditions [17, 18]. Irrigation also increases cooling and ET [18, 19]. However, some researchers consider non-irrigated green roofs to be more sustainable than irrigated green roofs due to their diminished requirements for construction materials, maintenance, and potable water use [18]. Furthermore, many studies have found that irrigation reduces green roof stormwater retention [12, 18, 20–22]. Li and Babcock [5] submit that because *Sedum* spp. are drought-tolerant, the lower moisture content of non-irrigated substrate planted with *Sedum* spp. will absorb more stormwater and compensate for lower plant transpiration. However, a major limitation of existing studies on green roof performance is that very few cover a period longer than one year [16, 23]. One of this study’s contributions is that it examines a four-year timeframe.

**Fig 1:**
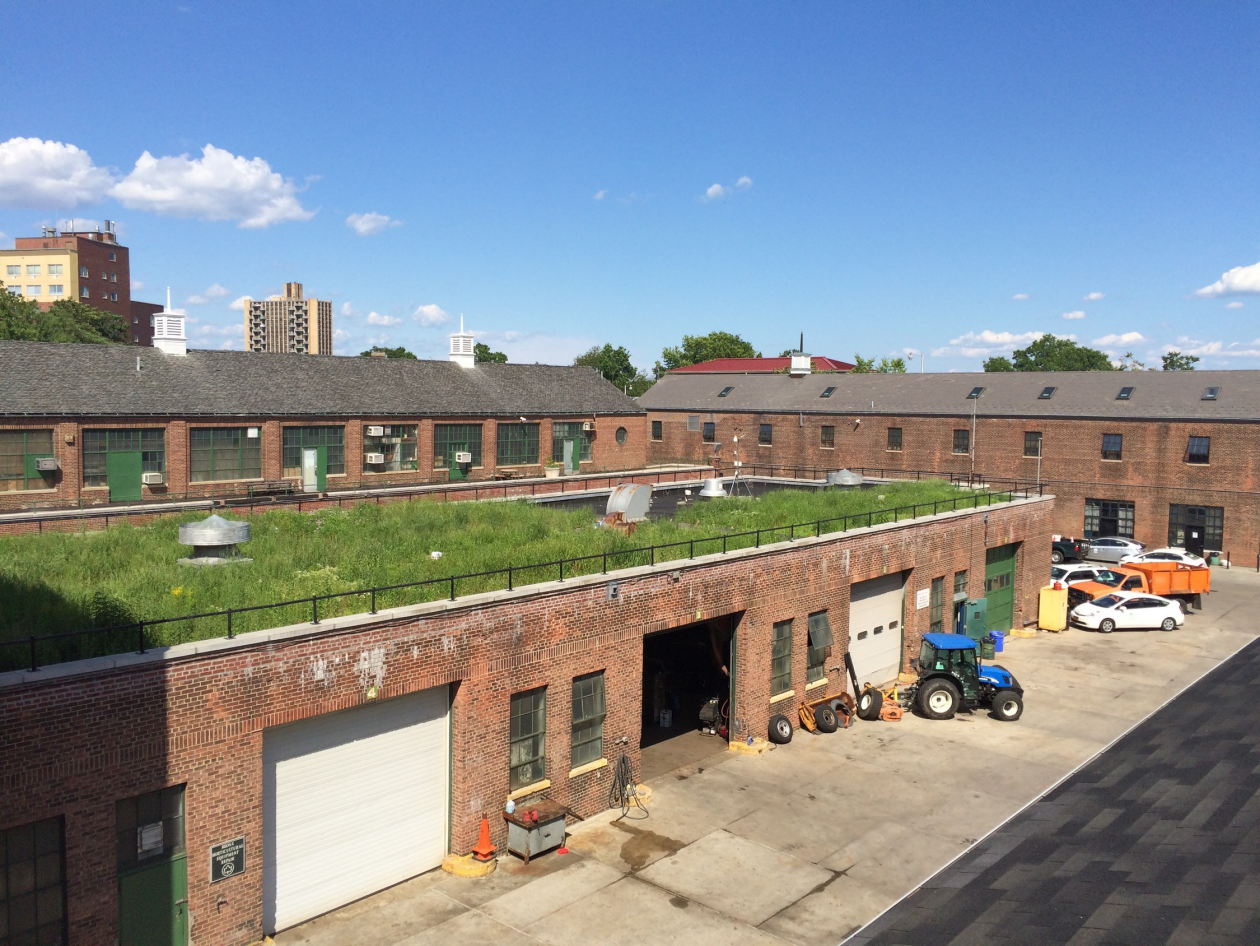
Ranaqua. The green roof is located above a NYC Parks auto garage in the Bronx, NYC.

The main objective of this study is to investigate differences in stormwater performance between an irrigated green roof planted with vegetation native to the NYC area and a non-irrigated green roof planted with *Sedum* spp. This study aims to do so through the analysis of four years of rainfall and runoff data collected from two full-scale green roofs, one irrigated native roof (Ranaqua; Fig 1) and a non-irrigated *Sedum* spp. roof (USPS; Fig 3), located in NYC. NYC shares the cold climate of the Northeastern U.S. but has hot summers (> 22°C) with little variation in rainfall among seasons [24]. NYC has an average annual rainfall of 1.3 m and an average daily temperature of 12.9°C [6].

Seasonal stormwater retention is compared across both roofs, and an empirical relationship between rainfall and runoff is developed to compare stormwater retention as a function of storm sizes. This study then distinguishes ET for native vegetation and *Sedum* spp. by determining crop coefficients using local climate and substrate moisture data. The main study objective can be broken down into two goals:

1. Compare stormwater retention between an irrigated green roof planted with NYC- native vegetation and a non-irrigated green roof with drought-tolerant vegetation;
2. Provide crop coefficients that distinguish evapotranspiration for NYC-native vegetation and drought-tolerant vegetation.

## Materials and methods

### Green roofs and instrumentation

#### Ranaqua green roof

The study green roof constructed with plants native to the NYC region (Fig 1) was located at Ranaqua, the Bronx headquarters of the New York City Department of Parks & Recreation (NYC Parks), at 40°50’50”N, 73°52’13”W. This second-floor roof, which has a total area of 638 m^2^_,_ will hereafter be referred to as “Ranaqua.” In October 2012, NYC Parks planted native vegetation on three quadrants of the roof, each draining a separate area and pitched at a 1.0% slope toward each quadrant’s respective drain. The fourth quandrant was left bare. Non-vegetated walkways that are 61 cm wide surround each quadrant and each rooftop HVAC unit with Delaware river gravel. Aluminum barriers were installed with roofing cement along high points to ensure that no stormwater runoff flows between quadrants.

**Fig 2:**
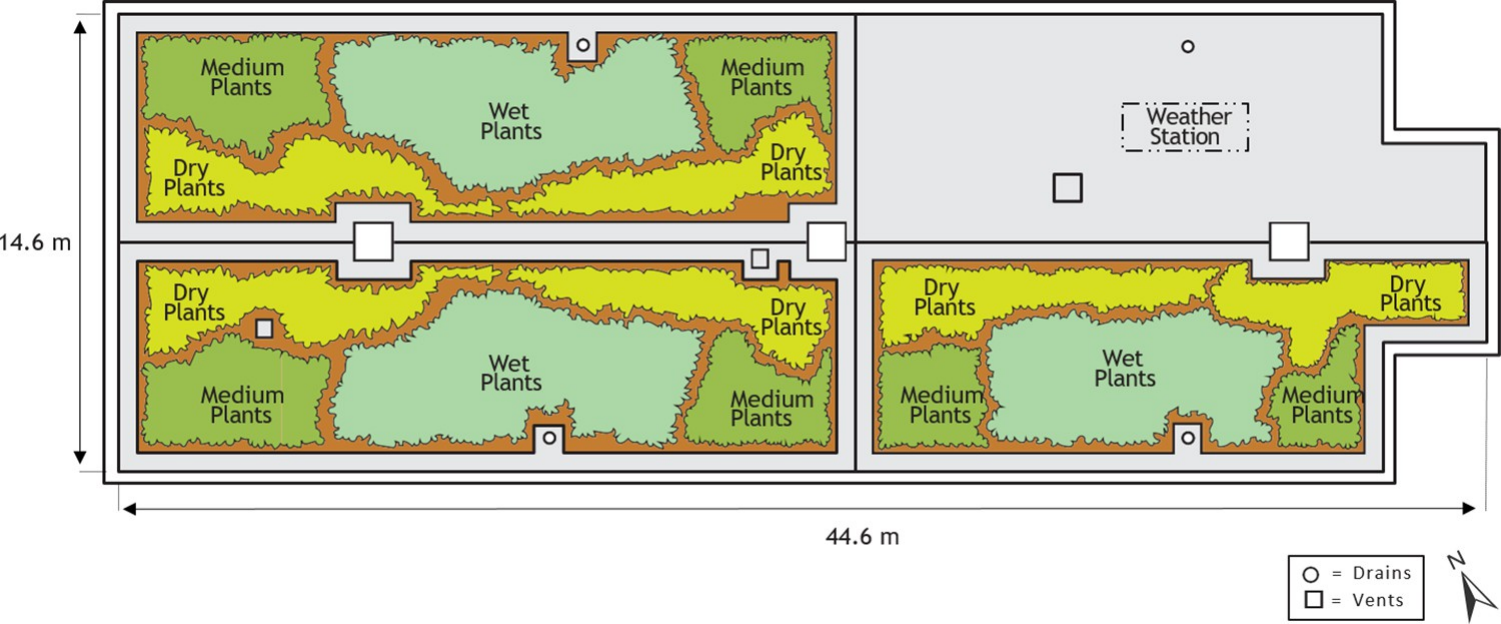
Wet, medium moisture, and dry planting zones on the Ranaqua green roof.

Each vegetated quadrant at Ranaqua was further divided into three zones based on plant irrigation requirements (Fig 2). Landscape architects at NYC Parks designed this arrangement to select from a diverse palette of plants, all native to the area within 50 miles of Manhattan, and with varying water requirements. A “wet” zone was planted with *Symphyotrichum novae-angliae*, *Verbena hastata*, *Scirpus cyperinus*, *Carex vulpinoidea Michx*., *Dichanthelium clandestinum*, *Elymus virginicus*, *Eupatorum maculatum*, *Eupatorium perfoliatum*, *Euthamia graminifolia*, *Helenium autumnale*, *Juncus tenuis*, *Monarda fitulosa*, *Eragrostis spectabilis*, *Rubus Flagellaris*, *Parthenocissus quinquefolia*, a “medium” zone was planted with *Eupatorium serotinum*, *Pycnanthemum tenuifolium*, *Pycnanthemum virginianum*, *Solidago juncea*, *Symphyotrichum laeve*, *Symphyotrichum pilosum*, and a “dry” zone was planted with *Andropogon virginicus*, *Danthonia spicata*, *Euthamia tenuifolia*, *Panicum virgatum*, *Solidago nemoralis*, *Sorghastrum nutans*, *Tridens flavus*, *Schizachyrium scoparium*. Plants were selected from native communities expected to resemble the windy and limited-soil depth conditions found on green roofs. Many of these plants are native to either the rocky summit grassland community found on mountains throughout New York State, or the Hempstead Plains grassland community found on Long Island [25].

Irrigation was applied almost daily during summer months (June, July, and August) at a maximum of 19 mm/week in the wet zone, 13 mm/week in the medium zone, and 8 mm/week in the dry zone. Irrigation was also applied when volumetric moisture content readings from a 10HS Decagon Devices soil moisture sensor installed in each zone dropped beneath 14%, which was the Allowable Depletion [26] recommended for the roof by the manufacturer of the irrigation equipment. Further details on the operation of the irrigation system for the roof can be found in Shetty [27].

Because the three vegetated quadrants had virtually identical runoff for 250 storms over the four-year monitoring period [27], the analysis of the Ranaqua green roof refers to data from a single quadrant for the remainder of this paper. This quadrant had a watershed area of 180 m^2^ and contained 127 mm of a growing substrate blend known as Norlite Expanded Shale aggregate mixed with We Care Compost, with a reported organic content of 5% to 8% and a maximum media water retention of 38%. Stormwater runoff from this quadrant drained into a 1,893 L (500 gallons) cistern located in an auto garage beneath the green roof. An HRXL-Max- Sonar-WR #MB7360 MaxBotix Inc. acoustical sensor recorded the water level in the cistern, allowing determination of runoff during storms based on changes in the stored volume. Overflow from the cistern was measured with a V-notch weir so runoff that exceeded the cistern’s storage capacity could also be determined.

Local climate data for Ranaqua was measured with an Onset Hobo U30 weather station located on the non-vegetated quadrant. The weather station recorded data from a TR-525i Texas Electronics tipping bucket rain gage and an S-THB- M002 Onset temperature and relative humidity sensor.

#### USPS green roof

**Fig 3:**
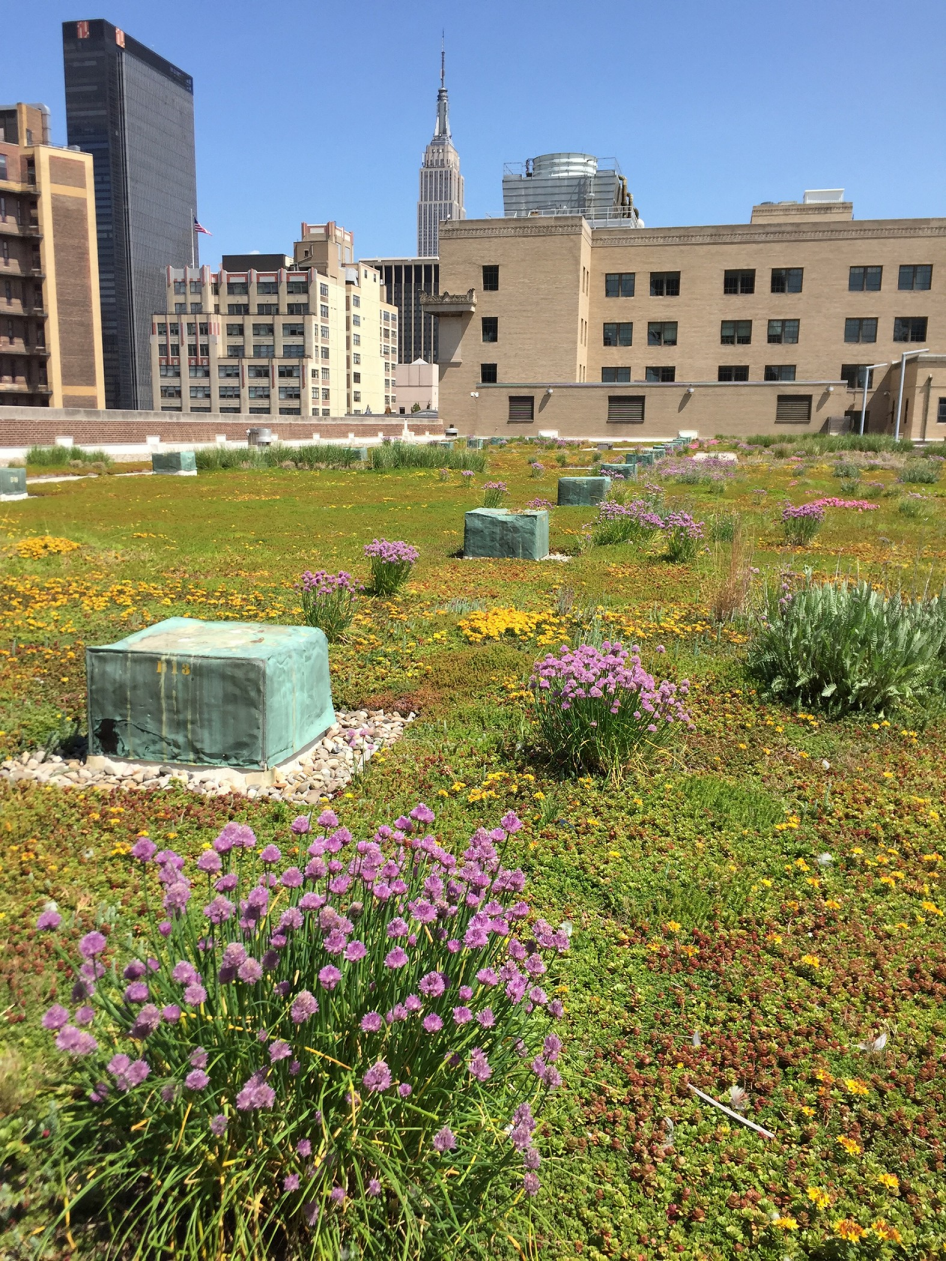
USPS. The green roof is located above the US Post Office’s Morgan Processing and Distribution Center in Manhattan, NYC.

The hydrological performance of the Ranaqua green roof was compared to the hydrological performance of a non-irrigated *Sedum* spp. green roof (Fig 3) located on the seventh floor of the US Postal Service Morgan Processing and Distribution Center, referred to as USPS, at 40°45’4”N, 73°59’60”W. This green roof, which is approximately 10,000 m^2^, was installed by Tecta Green in 2009 and has base slopes <2% toward rooftop drains. The roof has an expanded shale based growing substrate known as Skyland Rooflite extensive blend, with 8% reported organic content. The maximum media water retention, as analyzed by the Agricultural Analytical Services Laboratory at Pennsylvania State University, varies from 35% to 65%. The majority (97%) of the 390 m^2^ monitored watershed area on USPS has a 100 mm deep growing substrate and was planted with drought tolerant *Sedum* spp. However, 3% of the monitored watershed area also contains a single 200 mm deep berm, 2m x 6m in area that was planted with native vegetation.

The USPS green roof was instrumented with an Onset Hobo U30 weather station that recorded environmental conditions from an S-RGB-M002 tipping bucket rain gage and a THB-M002 2-bit air temperature/relative humidity sensor. The weather station was located on a vegetated portion of the roof. Roof runoff was recorded from a custom-designed weir device. The custom weir was designed to fit above the existing roof drain and included a V-notch weir and a Senix TSPC-30S1 ultrasonic distance sensor. An S-SMC-M005 EC-5 soil moisture sensor was used to measure substrate moisture content. Carson et al. [6] contains a full description of the USPS green roof, including instrumentation set-up and monitoring protocols.

The two green roofs are located 15.2 km away from each other. Instrumentation on both the Ranaqua and USPS roofs had a five-minute logging frequency. No permits were required to access the field sites, as both the US Postal Service and NYC Parks partnered with the research team directly for this study.

### Analysis

#### Storm event definition

The Ranaqua and USPS green roofs were monitored between November 1, 2013 and December 31, 2017. The total depth of each recorded rooftop storm event during this monitoring period was calculated using a minimum six-hour dry period to separate storm events [6, 28, 29]. This storm separation resulted in 433 storms at Ranaqua and 431 storms at USPS. Quality control of storms suitable for analysis were the same as those adopted by Shetty [27] and Carson et al. [6]. Storms were eliminated for which blank sensor readings indicated sensor errors (14 storms at Ranaqua, 1 at USPS), and where freezing temperatures indicated that precipitation could be in the form of snow (45 storms at Ranaqua, 30 at USPS). Storms were also eliminated for which the runoff depth exceeded the rainfall depth (77 storms at Ranaqua, 144 at USPS), which occurred when peak runoff rates resulted in unreliable readings due to turbulence within the runoff chambers. At Ranaqua, 47 storms were removed, because the cisterns had not yet emptied from previous storms, thus preventing a clear measurement of runoff depth. These elimination criteria resulted in 250 storms at Ranaqua and 256 storms at USPS that were considered suitable for analysis. Table 1 provides a summary of the number of storms used for analysis on each green roof, as well as details on storm groupings by event size category and season.

**Table 1:** Number of studied storm events at each green roof by size grouping and season, with seasons beginning June 21, September 21, December 21 and March 21.

	Spring	Summer	Fall	Winter	Total
**Ranaqua**	**81**	**60**	**66**	**43**	**250**
< 5 mm	51	23	33	19	126
5–10 mm	10	16	13	11	50
10–25 mm	11	12	9	5	37
> 25 mm	9	9	11	8	37
**USPS**	**92**	**73**	**48**	**43**	**256**
< 5 mm	51	32	21	19	123
5–10 mm	14	19	7	8	48
10–25 mm	16	16	11	14	57
> 25 mm	11	6	9	2	28

#### Stormwater retention

Observations of rainfall and runoff during the monitoring period were used to calculate stormwater retention with Eq (1):1

$$Stormwater\;retention\;(\%)=\frac{rainfall-runoff}{rainfall}$$ (1)

To estimate stormwater retention rates over time, Characteristic Runoff Equations (CREs) were developed for both green roofs. CREs are regression equations that allow one to estimate runoff depth (runoff per green roof watershed area) as a function of rainfall depth. They were calculated by fitting quadratic polynomials between measured rainfall and runoff depths [6, 30]. Storms that produced no runoff were excluded to avoid the estimation of negative runoff that is generated by CREs for small rainfall events [6], which resulted in 187 storms at the Ranaqua green roof and 164 at the USPS green roof that were used to obtain the CREs.

The CREs were then applied to historical climate data maintained by the National Oceanic and Atmospheric Administration National Climate Data Center (www.ncdc.noaa.gov). A rain gage at LaGuardia International Airport was selected for this analysis due to the availability of long term rainfall data at this gage, as well as the gage’s central location, which is approximately 10 km from each green roof. Using a minimum six-hour dry period to separate storm events, 4120 storms were measured at LaGuardia from March 1977 to March 2017, a 40-year period [6, 31].

For each of the 4120 storms recorded at LaGuardia (LGA) over the 40-year period, measured rainfall data at LGA was input into the respective CREs for each roof to calculate an annual estimate for runoff at each roof. Runoff was set to zero for rainfall less than the x-intercept values of the CREs [6]. With the measured cumulative rainfall and the estimated runoff depths summed for each of the 40 years, annual stormwater retention was then determined with Eq (1).

#### Evapotranspiration

Evapotranspiration (ET), in dispelling stormwater from green roofs, regenerates their stormwater retention capacity in between storm events. Total evapotranspiration for both green roofs was estimated with a water balance:

ET=Rainfall+Irrigation–Runoff–changeinsubstratemoisture (2)

This equation takes into account intercepted water (i.e. water that lands on vegetation) that eventually evaporates.

Unlike stormwater retention data, ET data was only able to be collected for a two-year period, November 18, 2013 to December 16, 2015, due to logistical concerns. Using Eq (2), the change in substrate moisture and total rainfall, runoff, and irrigation were input from November 18, 2013 to December 16, 2015, after removing the month of July, 2015 due to equipment failure at the Ranaqua green roof.

Once ET was estimated, crop coefficients (a measure of the evapotranspiration capacity of the green roof plants’) were estimated over the two year (728-day) period for both roofs with a soil moisture extraction function [10, 32, 33]:

$$ET\;=\;K_{c}*\sum_{n\;=\;1}^{728}\left(PET*\frac{\theta }{\theta_{max}}\right)$$ (3)

where PET denotes daily potential evapotranspiration, θ denotes the daily average substrate moisture value and θ_max_ denotes maximum water storage capacity for the substrate type. These values were summed over the two year period to solve for the crop coefficient K_c_.

This study used the Hargreaves and Samani equation [34] to model PET in Eq (3), due to its simplicity and accuracy [29, 35, 36]. Using the Hargreaves and Samani equation, PET was calculated using a daily time period [37] via the open-source software R v. 3.1.3 evapotranspiration package [38].

A more traditional method of finding crop coefficients was also performed at both green roofs, by dividing ET by PET. This simpler form is reported in many studies [31, 39] and may be useful to researchers who do not have soil moisture data. However, because soil moisture is a significant influence on evapotranspiration rates, Eq (3) may provide a more applicable and accurate model of green roof evapotranspiration [10, 29].

#### Statistical analysis

The non-parametric Wilcoxon rank-sum test was conducted in R v. 3.1.3 (The R Project for Statistical Computing, 2015) to compare annual retention and soil moisture at the two green roofs.

## Results

### Stormwater retention

**Fig 4:**
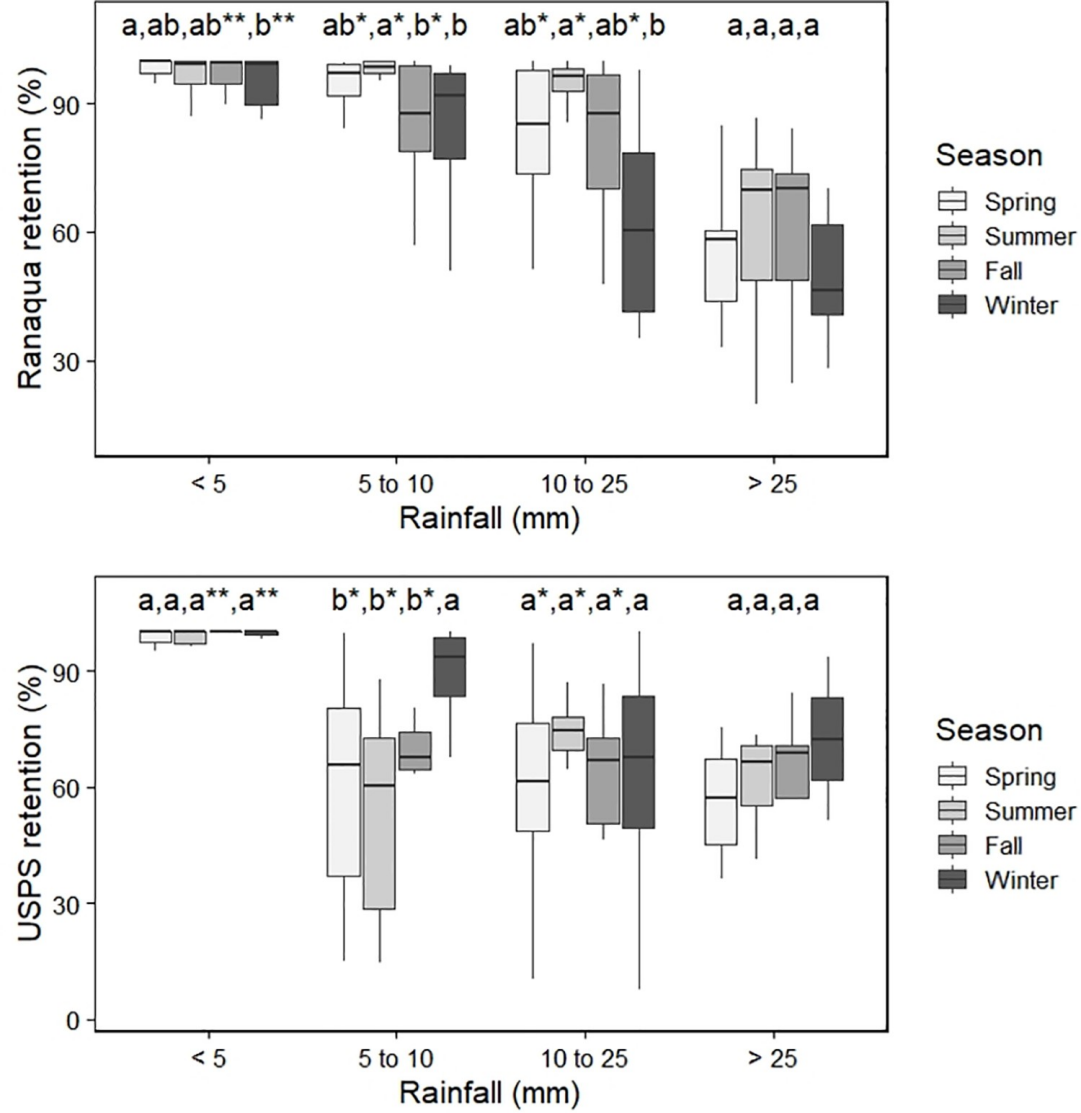
Seasonal box plots for different rainfall depths monitored from November 1, 2013 to December 31, 2017 at the green roofs (a) Ranaqua; (b) USPS. The letters represent statistically significant differences among seasons within a particular range of rainfall depth (two seasons that share a letter are not statistically different). * denotes groupings where Ranaqua is statistically greater than USPS, while** denotes groupings where USPS is greater than Ranaqua.

Stormwater retention was calculated for each event at both green roofs and plotted seasonally for four ranges of rainfall depths (Fig 4). Larger rainfall depths resulted in lower retention rates. In the smallest events (< 5mm), retention rates were nearly 100%, although Ranaqua had lower retention than USPS during the fall and winter. For intermediate storm sizes between 5 and 25 mm, Ranaqua had greater retention than USPS during the spring, summer, and fall. Ranaqua also demonstrated a seasonal trend, with greater retention rates in the summer than winter. The USPS green roof did not demonstrate a consistent seasonal trend for any storm size.

#### Characteristic runoff equations

**Fig 5:**
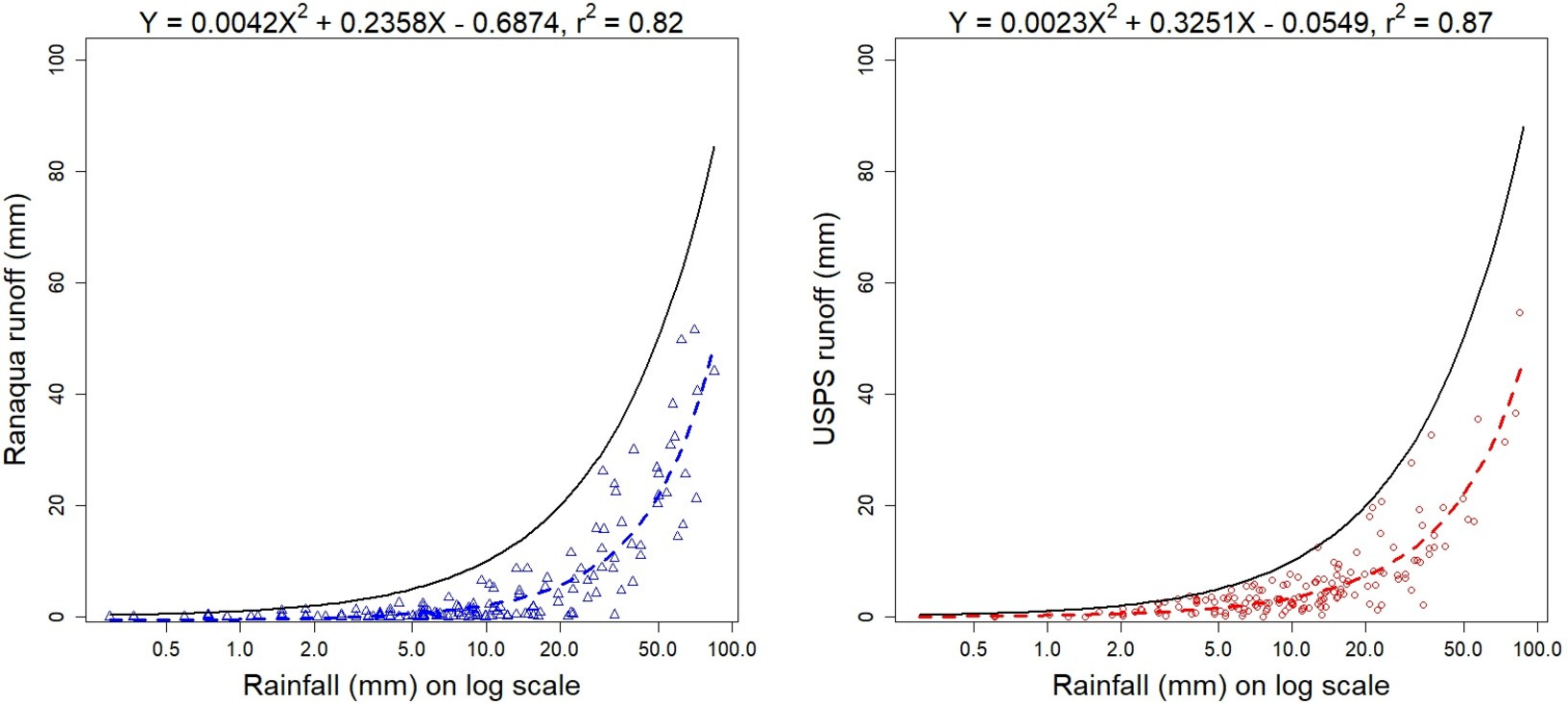
Rainfall versus runoff depths monitored between November 1, 2013 and December 31, 2017 for all storms with non-zero runoff. Characteristic Runoff Equations (CREs) are drawn as dotted lines for the green roofs (**a**) Ranaqua; (**b**) USPS. The black solid curves denote 1:1 agreement, representing hypothetical roofs where all rainfall becomes runoff.

The CREs for the USPS and Ranaqua green roofs are each presented with their respective plots of rainfall depth versus runoff depth in Fig 5 for all storms with non-zero runoff. While the estimation of runoff could be improved by considering factors such as the antecedent dry weather period before storms and potential evapotranspiration [31], the CREs explained 82% to 87% of all observed variation in runoff depth using rainfall depth alone.

The CRE for Ranaqua appears further deflected away from the 1:1 curve than USPS, indicating that Ranaqua attenuates more stormwater runoff.

#### Multi-year projections

**Fig 6:**
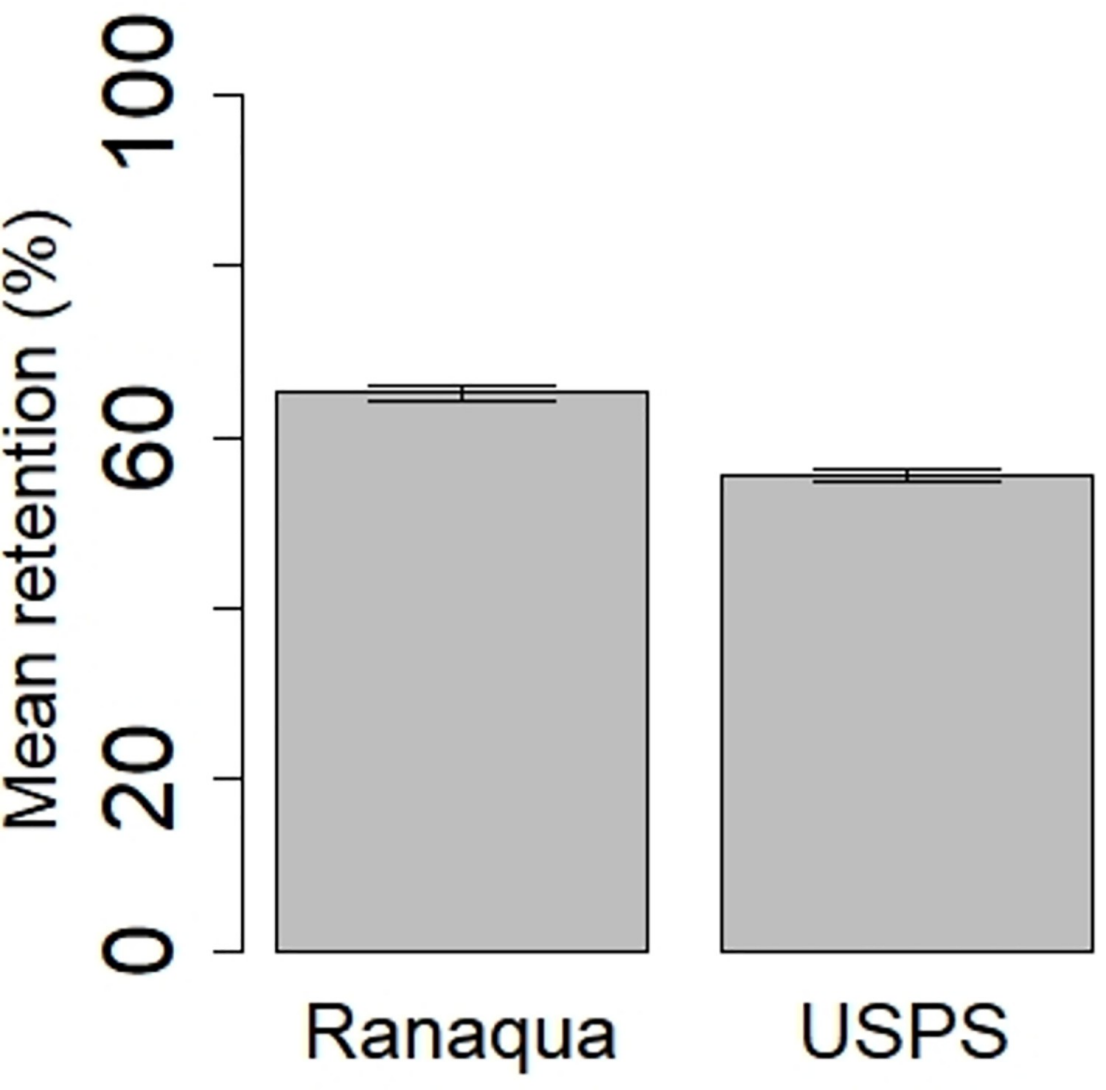
Estimated annual retention means ± standard error.

Each CRE was used to estimate annual retention using forty years of measured rainfall (Fig 6). Based on the multi-year projections, the Ranaqua green roof had a higher mean annual retention (64%) compared to USPS (54%), n = 40, *p* < 0.0001.

### Evapotranspiration

**Fig 7:**
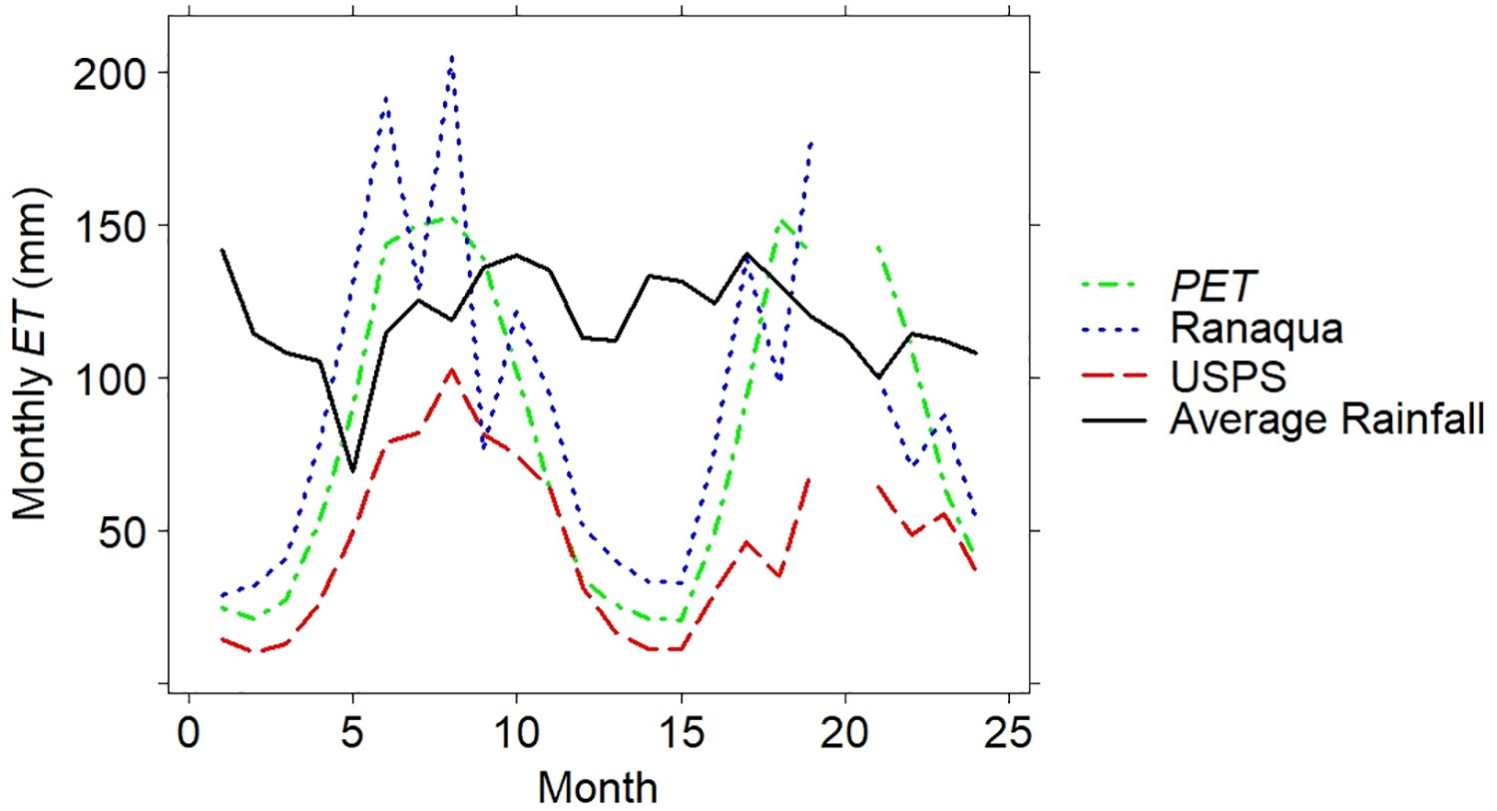
Monthly potential evapotranspiration (PET) and monthly evapotranspiration (ET) from both green roofs. Data are from December 2013 to November 2015, omitting July, 2015. Monthly rainfall from the Ranaqua weather station is drawn as a 6-month moving average in order to reduce noise from rainfall variability and more clearly demonstrate when PET and ET surpass average rainfall.

Using Eq (2), total evapotranspiration was determined for the two-year timeframe, leading to a calculation of 2097 mm for the native Ranaqua green roof and 1073 mm for the *Sedum* spp. green roof at USPS. Inputting this data into Eq (3), the estimated crop coefficient was 3.32 for Ranaqua and 1.63 for USPS. Similarly, when dividing evapotranspiration by potential evapotranspiration, the estimated crop coefficients were 1.13 (Ranaqua) and 0.57 (USPS). Finally, in order to parse out our original total ET number into a month-to-month basis, the estimated crop coefficients were re-applied to the soil moisture extraction function (Eq (3)) to estimate monthly ET. Fig 7 presents this information for each roof, along with monthly PET.

Since monthly rainfall varies significantly, the monthly rainfall at the Ranaqua weather station is presented as a 6-month moving average. As Fig 7 indicates, the Ranaqua green roof had much greater ET than USPS, and even frequently exceeded PET, while ET at USPS always remained less than PET.

All data used in this study are posted in the Supporting Information section S1–S3 Datasets.

## Discussion

This study indicates that conventional *Sedum* spp. green roofs might not manage as much stormwater as irrigated green roofs that are planted with native vegetation in the New York City climate region. In this study, the crop coefficients that were calculated demonstrate reduced ET in *Sedum* spp. (1.63, 0.57) compared to native plants (3.32, 1.13).

Ranaqua had greater annual stormwater retention (64%) compared to the more traditional *Sedum* spp. green roof (54%), based on a 40-yr projection from four years of monitoring data. This challenges the extent to which irrigation reduces retention, as suggested by Schroll et al. [21], Van Mechelen et al. [18], Volder and Dvorak [20], Whittinghill et al. [12], and Hill et al. [22]. While it could be that the above studies’ disparate results are due to the differing soil types used, it is believed more likely that Ranaqua’s greater stormwater management should be attributed to increased canopy interception and ET provided by its robust native vegetation. Irrigation likely contributed to both healthier vegetation and increased plant biomass. Indeed, the average leaf area index for *Sedum* plants used at USPS was found to be 2.8 [40–43], while the leaf area index for grasses and forbs used at Ranaqua averaged 3.7 [44–48] and 3.9 [49, 50] respectively.

Improved stormwater performance due to the presence of greater plant biomass thus overrode any reduction in stormwater retention due to diminished substrate storage because of irrigation. This finding directly contradicts Li and Babcock [5], who suggest that the lower substrate moisture of green roofs planted with *Sedum* spp. compensates for decreased water uptake by the vegetation. The findings of this study indicate that increased transpiration and canopy interception that occurs on irrigated, natively-planted green roofs leads to better stormwater management than non-irrigated green roofs planted with *Sedum* spp.

**Fig 8:**
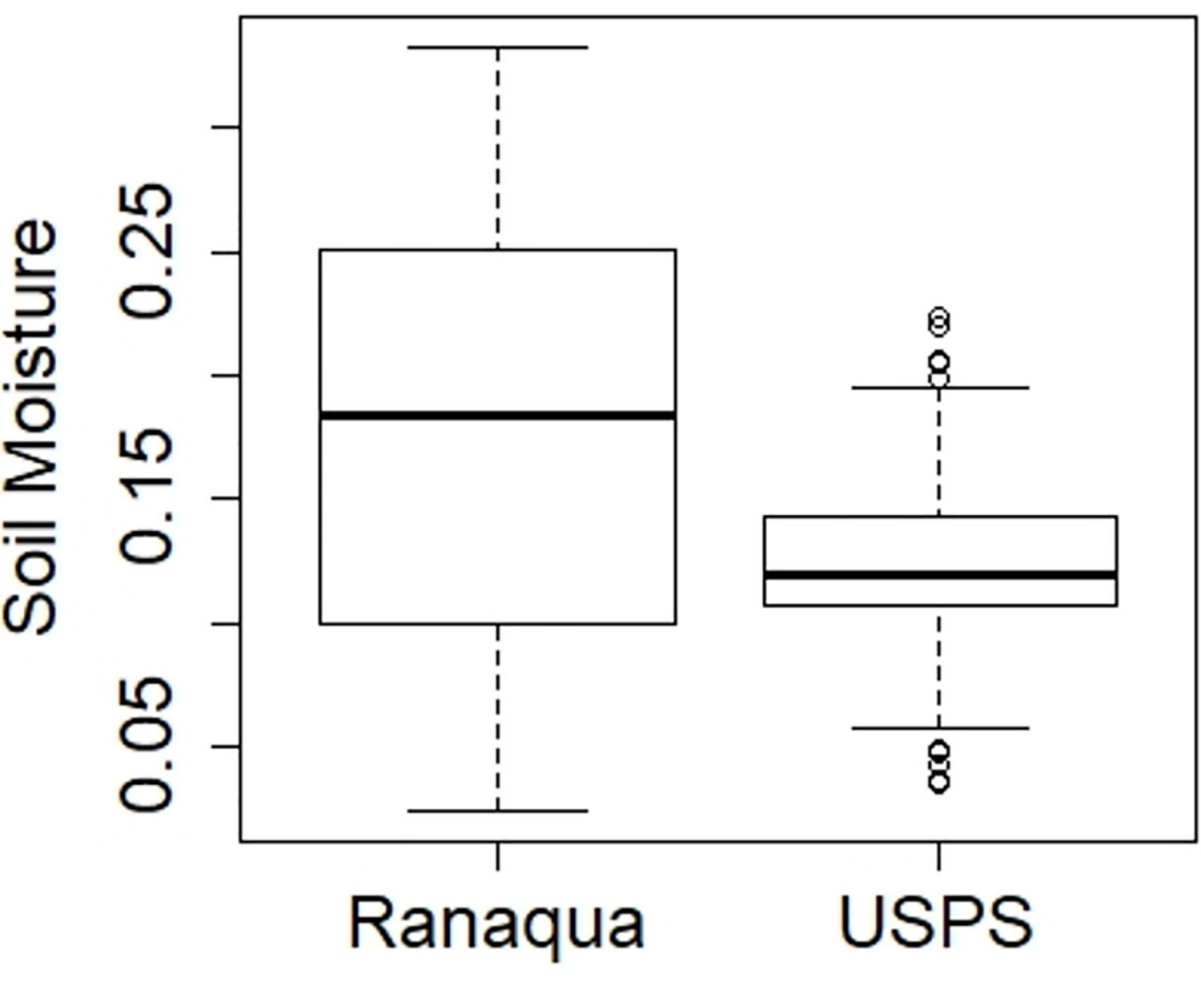
Soil Moisture at the beginning of rainfall events.

The sole event size category where the USPS green roof outperformed the Ranaqua green roof occurred during “small” storms (<5 mm) (Fig 4). For small storms, green roof substrates generally remain unsaturated, and runoff from green roofs is thought to be influenced by rainfall on non-vegetated surfaces [6]. In fact, the rooftop drains at Ranaqua are located adjacent to the non-vegetated perimeter, while at USPS, flow pathway lenths from non-vegetated areas to the rooftop drains are longer, allowing extended opportunity for depression storage and evapotranspiration to remove rainwater before it enters the drain. In addition, irrigation at Ranaqua may have reduced substrate stormwater storage, causing some runoff even during small events. For small storms, the irrigation amount is a greater percentage of the rainfall depth, and therefore causes a greater decrease in percent retention. Indeed, soil moisture at the beginning of rainfall events was greater (*p* < 0.0001) at Ranaqua than for USPS, averaging 18% at Ranaqua as opposed to 12% at USPS (Fig 8).

**Fig 9:**
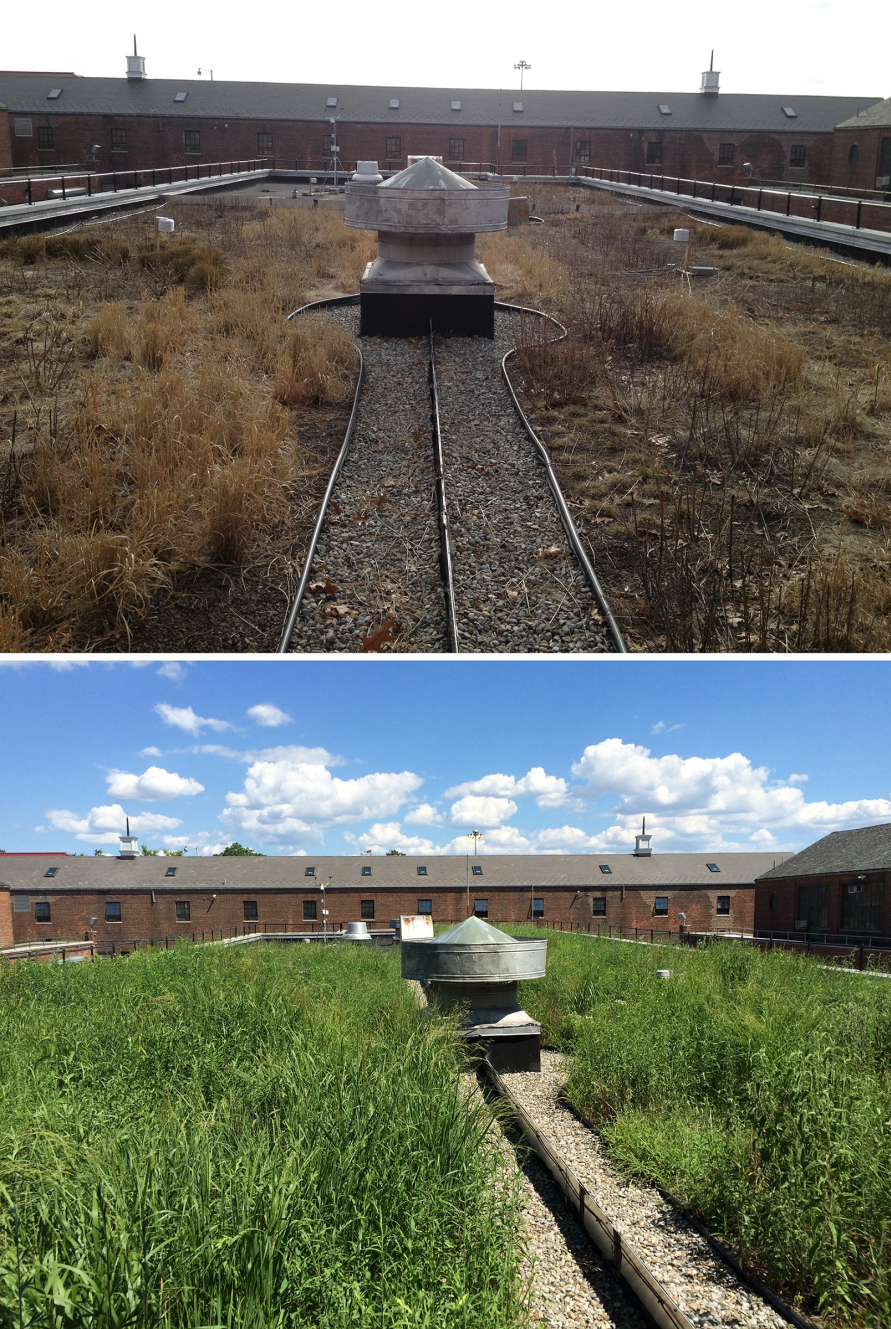
Ranaqua green roof during the (a) winter; (b) summer.

Warmer summer temperatures have been generally associated with greater rates of green roof stormwater retention [2, 10, 15, 30, 51]. Greater seasonal variation (Fig 4) is associated with thin substrate and large storms [21, 31]. Despite its thicker substrate (127 mm) compared to USPS (100 mm), Ranaqua followed a seasonal trend for storms between 5 and 25 mm, with greater retention in the summer than winter This trend was likely caused by the annual growth of native grasses, which have been found to display a greater seasonal effect than *Sedum* spp. on stormwater retention [16]. During the winter, the dead stems of native grasses are cut and removed from Ranaqua (Fig 9A). The removal of the grass cover reduced canopy interception during the winter and spring until the next growing season.

The greater crop coefficients found for Ranaqua than for the roof vegetated with *Sedum* spp., highlights the capability of NYC-native vegetative cover to transpire more stormwater given certain climate and soil moisture conditions. Crop coefficients for USPS were in the general range found by other studies modeling green roofs planted with *Sedum* spp., as the 0.57 found at USPS (ET/PET) is consistent with the 0.8 average found by Harper et al., and the 0.27 to 0.79 range found by Starry [39, 52, 53]. The crop coefficient (ET/PET) was 1.13 at Ranaqua, which is greater than the 0.7 to 0.8 maximums typically reported in prior studies of green roof vegetation, although crop coefficients as high as 3.25 have been found for brief periods of summer [54]. The crop coefficients calculated in previous studies included a wide range of soil moisture, including well-watered (crop coefficients ranged from 0.4 to 0.52) and water-stressed (crop coefficients ranged from 0.1 to 0.34) [54]. The irrigation and resulting wet substrate at Ranaqua is one reason for the larger crop coefficients. The greater crop coefficients at Ranaqua may also represent the capability of native vegetation to improve stormwater retention through canopy interception and ET when native plants develop substantial canopy biomass, possibly due to irrigation.

Not only did Ranaqua’s native vegetation have greater ET than USPS (Fig 7), but ET at Ranaqua even exceeded PET, while ET at USPS remained less than PET. With greater crop coefficients and the presence of additional water from irrigation, ET at Ranaqua surpassed the average rainfall during the summer months. USPS did not provide as much ET due to the lack of irrigation, and due to *Sedum* spp., which transpire more slowly, and do not develop as much biomass as the NYC-native vegetation. The smaller amount of biomass may lead to lower evapotranspiration and canopy interception for *Sedum* spp.

This study benefits from four years of observed hydrological data from two full-scale green roofs. Many monitoring studies contain shorter monitoring periods, which can produce contradictory results [23]. In fact, few studies even last one full year [16]. Variation in stormwater retention among green roofs reported in the literature may be largely due to varying durations of monitoring, rather than actual performance [6, 28].

## Limitations of the study

This study was somewhat limited however by the different characteristics of the two green roofs, such as the varying substrate depths (Table 2). As a result, the effects of vegetation and irrigation on water retention could not be completely isolated. However, full-scale green roofs that are identical except for a single independent variable do not exist. All published field studies on full-scale rather than pilot-scale green roofs have multiple differences. And while pilot-scale studies using elevated test boxes or similar modules can test a single independent variable, it is uncertain how accurately they forecast full-scale performance [6]. In essence, this study does not test one variable, but two completely different green roof designs. The reason that *Sedum* spp. green roofs are sometimes favored is due to the fact that they do not require irrigation and deep substrate, whereas a green roof planted with these native species would not succeed without both irrigation and deep substrate. The bottom line remains that this study found that the performance of green roofs planted with NYC-native species was overall superior to a *Sedum* spp. planted green roof’s, despite the former requiring irrigation.

**Table 2:** Confounding factors.

	Ranaqua	USPS	Possible Influence on Stormwater Retention
**Media Type**	Expanded Shale Aggregate	Expanded Shale Aggregate	None
**Media Organic Percentage**	5 to 8	8	Greater retention by USPS
**Media Depth (mm)**	127 mm	100 mm	Greater retention by Ranaqua
**Average daily temperature**	11.17°C	11.67°C	Greater retention by USPS
**Average daily relative humidity (%)**	65.33	68.44	Greater retention by Ranaqua
**Average daily wind speed (m/s)**	1.01	1.39	Greater retention by USPS

For example, although these two roofs use slightly different soil compositions, with Ranaqua built with Norlite Expanded Shale aggregate, and USPS with Skyland Rooflite, this minor difference is immaterial, as both soils have an expanded shale aggregate base. Thus, media type would not be an influential factor in the different stormwater retention behaviors observed between the two roofs.

Along these same lines, there might be a difference in media organic percentage between the two studied roofs, with USPS having a higher percentage of organic matter (OM) that Ranaqua (8% versus 5–8%, respectively). However, higher OM content is positively associated with greater water retention [55], and thus better retention performance. This thus eliminates OM content as a potential confounding factor, as higher OM content in USPS would point to better stormwater retention for the USPS roof versus the Ranaqua roof, all other factors being equal.

Similarly, the slightly differing temperatures and relative humidity rates of the two sites could impact results, as green roof ET increases with increasing temperature and decreases with increasing relative humidity. The Ranaqua roof averaged a temperature of 11.17°C, with a relative humidity of 65.33%, while USPS averaged 11.67°C with a relative humidity of 68.44%. According to Marasco’s [56] models for green roof ET, the average daily ET based on these numbers would be equal, at 50.4mm per month. The higher temperature at USPS is thus counter-balanced by its higher relative humidity, so all other things being equal, temperature and relative humidity should not lead to differences in stormwater retention.

Furthermore, the Ranaqua roof is on the second floor, while the USPS roof is on the seventh floor, potentially impacting wind speed and therefore rooftop evapotranspiration. As Cascone [57] shows, wind speed has a positive correlation with green roof ET. The USPS roof averages a daily wind speed of 1.39 m/s, while Ranaqua has an average daily wind speed of 1.01, thus giving an estimated percentage difference between the roofs of 8%, in favor of USPS. Ranaqua’s superior performance thus is despite, not because of, wind speed’s impact on rooftop ET. The Supporting Information section includes temperature (S1 Fig), humidity (S2 Fig), and wind speed (S3 Fig) data for Ranaqua and USPS from December 2013 to May 2016, omitting July 2015.

Finally, although Ranaqua has a deeper substrate than USPS (127 mm versus 100 mm), most studies conclude that media depth does not significantly influence green roof stormwater retention. To wit: VanWoert et al. [58] studied 100 mm and 200 mm media depths and found that choice of media depth had no significant influence on green roof water retention, while Fassman-Beck et al. [28] similarly report no significant difference in runoff between mini-roofs with substrate depths of 100mm and 150 mm, respectively, and Wanielista et al. [59] noted a relative insignificance of substrate depth on stormwater retention. And while it is true that other studies do show some impact of deeper substrate on water retention, Ranaqua’s performance still exceeded what would have been expected due to an increase in substrate depth alone. Schultz et al. [60] found that a 50 mm increase in substrate depth resulted in a 10% increase in stormwater retention. Here however, the 27 mm difference in substrate depth between USPS and Ranaqua resulted in a 19% increase in stormwater retention. Other studies support this: for example, Stovin et al. [35] found that crop coefficients had a greater influence on stormwater retention than substrate depth, while Talebi et al. [61] performed a sensitivity analysis which revealed that vegetation type had a greater impact on the stormwater retention performance of green roofs than increases in substrate depth. In that study, increases in substrate depth from 80 mm to 220 mm increased stormwater retention by 2.4% to 8.8%, while in contrast, stormwater retention increased by 13.5% when high water use plants replaced low water use plants. And finally, Fassman-Beck et al. [28] even found that four different green roof substrate depths were equally effective in reducing stormwater runoff.

As a result, the greater stormwater retention at Ranaqua appears to be more due to higher crop coefficients than substrate depth. While some confounding factors, such as differences in average daily temperature and average RH between the roofs, balance each other out when it comes to potential influence on green roof stormwater retention, others, such as differences in average daily windspeed, point to greater retention by USPS. The only confounding factor that would lead to greater stormwater retention by Ranaqua is the greater depth of the Ranaqua substrate. It is estimated that, at maximum, this might account for 5% additional retention by Ranaqua. Given the 10% difference in retention observed, it is believed that the conclusions of the work still stand.

## Conclusions

Most extensive green roofs in temperate North America are non-irrigated and planted with drought-tolerant vegetation. The findings of this study suggest that irrigated green roofs planted with native vegetation may reduce stormwater runoff more than typical green roofs planted with *Sedum* spp. The observed crop coefficients demonstrate that native vegetation benefits not only biodiversity [2, 9, 62], but also stormwater management. This work may justify increased construction of “next generation” green roofs with native vegetation and irrigation in order to maximize stormwater retention.

## Supporting information

S1 Fig Temperature. (TIF)

S2 Fig Humidity. (TIF)

S3 Fig Wind speed. (TIF)

S1 Dataset Rain and runoff during monitoring period. (CSV)

S2 Dataset Multi-year projections. (CSV)

S3 Dataset Evapotranspiration. (CSV)
