# Supplementary figures and images for: Comparing the hydrological performance of an irrigated native vegetation green roof with a conventional Sedum spp. green roof in New York City
Source: PLoS One. 2022 Apr 20;17(4):e0266593. doi: 10.1371/journal.pone.0266593 (PMC9020694; doi:10.1371/journal.pone.0266593)

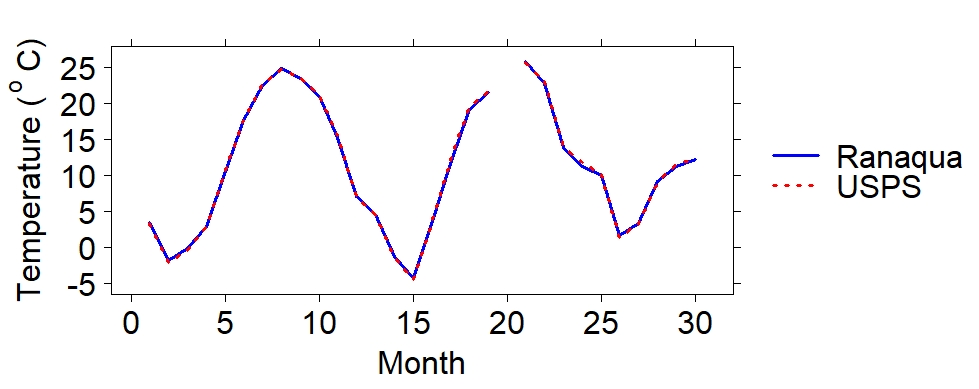

Supplement: S1 Fig — (TIF) [file pone.0266593.s001.tif]

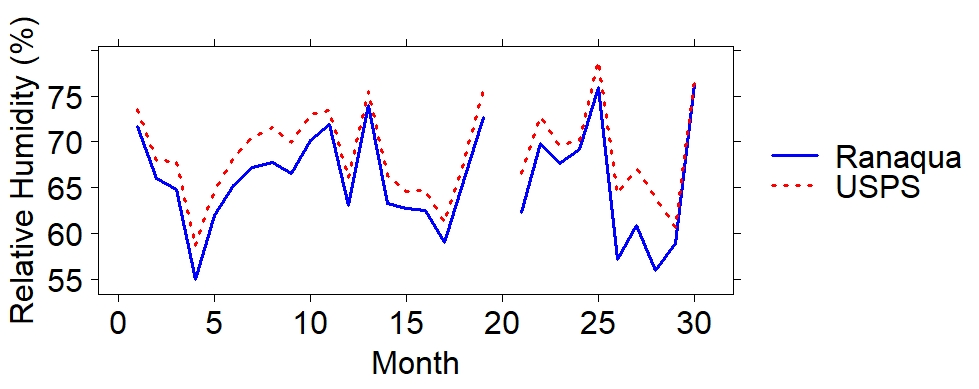

Supplement: S2 Fig — (TIF) [file pone.0266593.s002.tif]

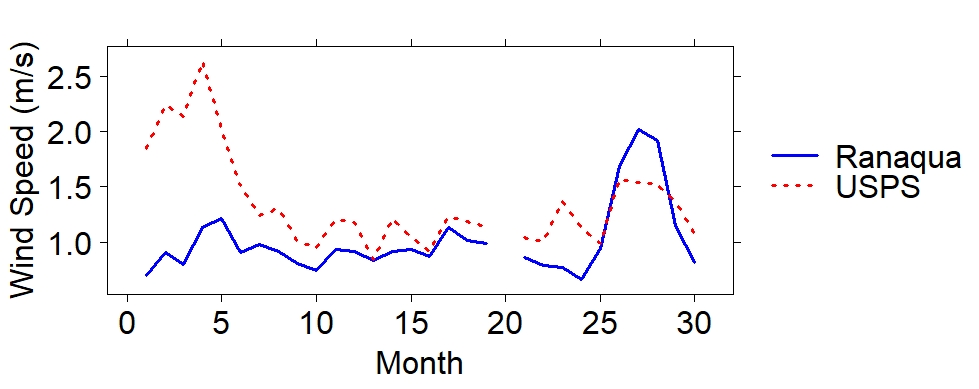

Supplement: S3 Fig — (TIF) [file pone.0266593.s003.tif]
